# Recent advances in the contribution of noncoding RNAs to cisplatin resistance in cervical cancer

**DOI:** 10.7717/peerj.9234

**Published:** 2020-05-29

**Authors:** Xin Wen, Shui Liu, Jiyao Sheng, Manhua Cui

**Affiliations:** The Second Hospital of Jilin University, Jilin University, Changchun, Jilin Province, China

**Keywords:** Cervical cancer, Cisplatin resistance, MicroRNA, Long non-coding RNA, Circular RNA, Noncoding RNAs

## Abstract

Cervical cancer (CC) remains a major disease burden on the female population worldwide. Chemotherapy with cisplatin (*cis*-diamminedichloroplatinum (II); CDDP) and related drugs are the main treatment option for CC; however, their efficacy is limited by the development of drug resistance. Noncoding RNAs (ncRNAs) have been found to play critical roles in numerous physiological and pathological cellular processes, including drug resistance of cancer cells. In this review, we describe some of the ncRNAs, including miRNAs, lncRNAs and circRNAs, that are involved in the sensitivity/resistance of CC to CDDP-based chemotherapy and discuss their mechanisms of action. We also describe some ncRNAs that could be therapeutic targets to improve the sensitivity of CC to CDDP-based chemotherapy.

## Introduction

There are 569,847 new cervical cancer (CC) diagnoses and 311,365 deaths from CC annually worldwide (Bray et al., 2018). Although the majority of CC patients with early stage disease can achieve a favorable prognosis with surgery and radiotherapy, patients with advanced or recurrent disease generally have poor outcomes (Pfaendler & Tewari, 2016; Waggoner, 2003). Nowadays, chemotherapy for CC is mainly used in three aspects: (1) neoadjuvant chemotherapy (NACT) to shrink the tumor before operation; (2) concurrent chemoradiotherapy (CCRT) for middle and advanced patients; (3) palliative chemotherapy for patients with recurrence or metastasis (Moore, 2008; Moore et al., 2010; Pfaendler & Tewari, 2016). Based on the guidelines of National Comprehensive Cancer Network (NCCN) for CC, Cisplatin (*cis*-diamminedichloroplatinum (II); CDDP) is considered as the first line drug of chemotherapy (National Comprehensive Cancer Network, 2020). However, the treatment response to CDDP varies, and the biggest obstacle to its efficacy is the development of drug resistance (Amable, 2016; Ko & Li, 2019). Thus, there is an urgent need to understand the underlying molecular mechanisms and identify strategies to overcome CDDP resistance in CC.

Noncoding RNAs (ncRNAs) make up the majority of the human transcriptome and are involved in many biological processes, including cell proliferation and differentiation, metabolism, the stress response, and apoptosis. Not surprisingly, abnormal expression and/or activity of ncRNAs has a profound effect on normal physiology and is associated with a number of pathologies, including cancer (Aalijahan & Ghorbian, 2019; Anastasiadou, Jacob & Slack, 2018; Djebali et al., 2012). According to the structural property, ncRNAs can be generally divided into linear ncRNAs and circular ncRNAs (circRNAs). And based on the length of linear ncRNAs, they consist of short ncRNAs such as microRNAs (miRNAs), siRNAs, piwi-interacting RNAs (piRNAs), snoRNAs, snRNAs, as well as long non-coding RNA (lncRNAs), including long intergenic ncRNAs, antisense RNAs (Chan & Tay, 2018; Peng & Calin, 2018).

Deregulation of ncRNAs can also affect the outcome of cancer treatment and allow tumors to acquire drug-resistant phenotypes (Corrà et al., 2018; Wang et al., 2019). An increasing number of studies has shown that ncRNAs play an essential role in CC (Chaichian et al., 2020; He et al., 2016; Hosseini et al., 2017; Sharma, Dua & Agarwal, 2014), and several classes of ncRNAs, such as miRNAs, lncRNAs and circRNAs, have been associated with CDDP resistance, making them important potential therapeutic targets (Matsui & Corey, 2017).

**Figure 1:**
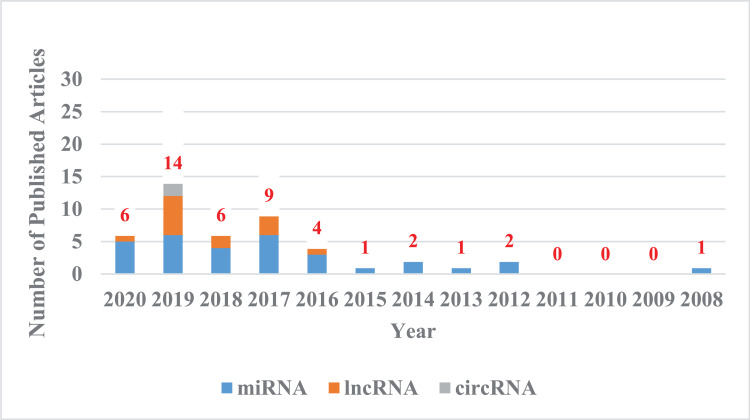
The trend chart of published articles related to the advanced of non-coding RNA in the CDDP/CC field over the years. We counted the articles related to the advanced of non-coding RNA in the CDDP/CC field in PubMed databases from their inception to April 8, 2020. Blue represents miRNA, orange represents lncRNA, and gray represents circRNA.

In this review, we summarize the current literature on the contribution of ncRNAs to CDDP resistance in CC (Fig. 1). We focus on studies examining the mechanisms of action of ncRNAs and the potential applications of ncRNAs in predicting CDDP sensitivity and improving chemotherapy regimens in CC, which might provide new therapeutic strategies for patients with CDDP-resistant CC.

## Survey Methodology

The authors conducted a systematic search with PubMed databases for articles published from their inception to April 8, 2020, to identify relevant studies published in English. The keywords for search include “cervical cancer”, “cisplatin”, “chemotherapy resistance”, “chemoresistance”, “chemosensitivity”, “chemotherapy sensitivity”, “microRNA”, “long noncoding RNA”, “non-coding RNA”, and “circular RNA”. Additional keywords, such as “carcinoma of uterine cervix”, “cervical neoplasia”, “cervix cancer”, “cervical carcinoma”, “carcinoma of cervix”, “circRNA”, “lncRNA”, “miRNA”, “ncRNA”, “noncoding RNA”, “cis-platinum”, “CDDP”, “DDP”, “drug-resistance”, “drug resistance”, and “sensitivity of chemotherapy”, were also used. Additional articles were identified by manual search of references found in the primary articles. The screened articles were used as references for this review.

## Mechanisms by Which ncRNAs Affect CDDP Resistance in CC

### Mechanisms of action of CDDP

**Figure 2:**
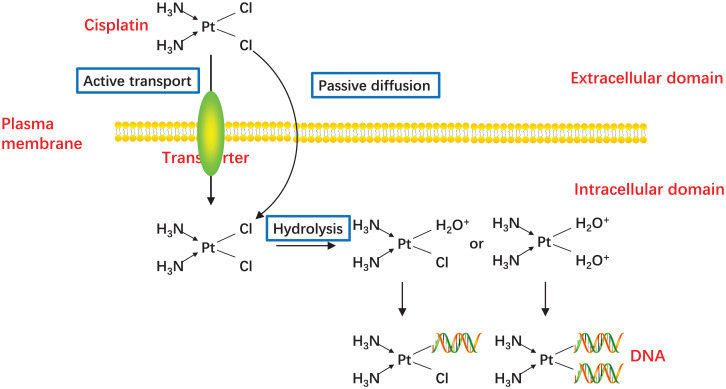
Activation of CDDP. CDDP can be passively diffused to tumor cells through the plasma membrane, and the transporter CTR1 is also responsible for CDDP uptake. The concentration of chloride in the blood is relatively high (approximately 100 mM), so the chemical properties of CDDP remain inactive. But the concentration of chloride in the cytoplasm is relatively low (approximately 4–20 mM), one or two of the chloride ligands are replaced by water ligands once CDDP enters into the cells. The hydrolyzed CDDP is highly reactive and mainly targets the nuclear DNA, then the DNA-platinum adducts cause DNA damages.

**Figure 3:**
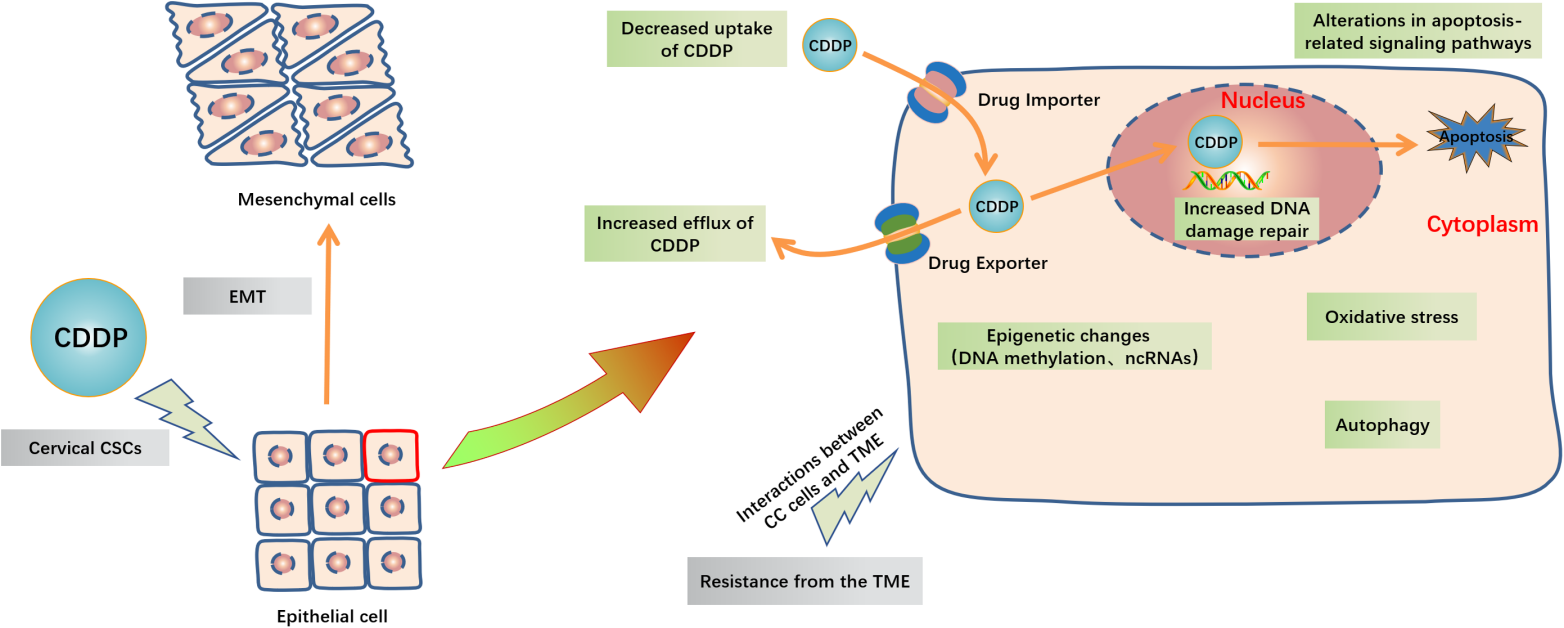
Schematic representation of intracellular and extracellular mechanisms of the development of CDDP resistance in CC. Inside the CC cells, decreased uptake of CDDP, increased efflux of CDDP, increased DNA damage repair, alterations in apoptosis-related signaling pathways, oxidative stress, autophagy and epigenetic changes including DNA methylation and ncRNAs are major mechanisms of cisplatin resistance. Outside the CC cells, EMT, cervical CSCs and TME like high cell density, hypoxia and acidity in tumor and the extracellular matrix interactions are also contribute to CDDP resistance. Abbreviation: TME: Tumor microenvironment; EMT: Epithelial-mesenchymal transition; GCL: Glutamate cysteine ligase; CSCs: Cancer stem cells.

In 1978, CDDP became the first platinum compound to be approved for cancer treatment in the United States (Kelland, 2007), and since then, CDDP has proven to be one of the most effective drugs for the treatment of advanced or recurrent CC (Small et al., 2017). CDDP enters cells mainly by passive diffusion, with contributions from active transport *via* copper transporters (Holzer, Manorek & Howell, 2006; Ishida et al., 2002). While CDDP itself is inactive, it becomes chemically active in the cytoplasm when one or both of its chlorine atoms are displace by water molecules (Fig. 2) (Galluzzi et al., 2014; Kelland, 2000). Hydrolyzed CDDP exerts its toxic effects by forming DNA-platinum adducts and by simultaneously initiating cellular self-defense systems through the activation or silencing of multiple genes (Eastman, 1987; Yang et al., 2006). DNA damage triggers a complex array of DNA damage response and repair pathways; however, if these processes cannot repair CDDP-induced DNA damage, replication and transcription is blocked, and the cells undergo cell cycle arrest and/or apoptosis (Brabec & Kasparkova, 2005; Dasari & Tchounwou, 2014). The diverse mechanisms by which cells respond to platinum-induced DNA damage provides a wealth of potential mechanisms for cancer cells to evade drug-induced death. Indeed, any factor that influences CDDP binding to DNA, the DNA damage response, or pathways leading to apoptosis could potentially lead to the emergence of drug resistance (Shen et al., 2012). The responsible mechanisms were summarized in Fig. 3.

### Mechanisms by which miRNAs affect CDDP resistance in CC

MiRNAs are short ncRNAs (generally <30 nucleotides) that play roles in post-transcriptional regulation (Lu & Rothenberg, 2018). In most instances, miRNAs act by binding to the 3′-untranslated region (3′-UTR) of the target mRNA, resulting in mRNA destabilization, degradation, or inhibition of translation. Various studies have reported abnormal miRNA expression in CC compared with normal cervical tissue (Juan et al., 2014; Lee et al., 2008), that could affect the proliferation and invasiveness of CC cells and thus their sensitivity to CDDP. Yang et al. (2016c) examined the relative expression of five miRNAs (miR-183, miR-182, miR-30a, miR-15b, and miR-16) and their potential target mRNAs in CDDP-resistant HeLa cell lines compared with their parent HeLa cell line, and they found that miR-182 and miR-15b were upregulated, while miR-30a was significantly downregulated, in the CDDP-resistant cells. Moreover, the mRNA targets of all these miRNAs were shown to be related to drug resistance.

Multiple miRNAs have been shown to influence CC cell growth, survival, and CDDP sensitivity *via* binding to the target mRNA 3′-UTR (Huang et al., 2017; Li et al., 2015; Sathyanarayanan, Chandrasekaran & Karunagaran, 2017; Yang et al., 2015a). For example, miR-217 binds to *KRAS* mRNA and directly targets it to mitigate the aggressiveness of CC cells (Yin & Ren, 2019). MiR-181a negatively regulates the expression of the apoptosis-related protein kinase Cδ (PRKCD) by targeting its mRNA for inhibiting apoptosis in CC cells (Chen et al., 2014). MiR-125a binds to STAT3 mRNA and inhibits its translation, resulting in downregulation of CC cell apoptosis and promotion of CDDP resistance (Fan et al., 2016). Finally, miR-144 targeting of the mRNA of LHX2, an oncogenic transcriptional regulator, was found to reverse the CDDP resistance of CC cells (Shi et al., 2019). In addition to these examples, miRNAs may affect CDDP sensitivity through the following mechanisms.

#### Promotion of drug uptake

Active transport of CDDP into and out of cells occurs *via* copper transporters, especially copper transporter protein 1 (CTR1, encoded by *SLC31A1*) (Bompiani et al., 2016; Kalayda, Wagner & Jaehde, 2012; Larson et al., 2009). Accordingly, CDDP-resistant CC cells have been found to express lower levels of CTR1 compared with their CDDP-sensitive counterparts (Zisowsky et al., 2007). Similarly, CTR1 knockdown significantly promotes the proliferation of CC cells in the presence of CDDP, while miR-130a binds directly to SLC31A1 mRNA, thereby regulating CTR1 protein expression (Feng et al., 2018). These results indicate that miRNAs can influence the sensitivity of CC cells to CDDP *via* regulation of copper transporter expression.

#### DNA repair

Intrachain and interchain DNA adduct formation by CDDP initiates DNA repair pathways, but a failure to re-establish genomic integrity can lead to cell cycle arrest or apoptosis. DNA repair is mediated predominantly by two processes: nucleotide excision repair and mismatch repair (Shen et al., 2012). Mismatch repair is regulated by several miRNAs, particularly miR-21 and miR-155 (Svrcek et al., 2013). Zhang et al. (2018a) examined gene polymorphisms in 165 chemotherapy-sensitive/resistant CC patients and found that rs1292037 (A > G) locus AG, GG, AG + GG and G allele in miR-21 gene was associated with CDDP sensitivity. Moreover, miR-155 expression also correlates positively with chemosensitivity to CDDP in human CaSki CC cells (Lei et al., 2012). These results indicated a possible link between miRNAs and CDDP sensitivity through the regulation of mismatch repair. Poly (ADP-ribose) polymerase 1 (PARP-1) is an important regulator of DNA repair and is negatively regulated by miR-7-5p, suggesting another mechanism by which miRNAs could contribute to CDDP resistance (Yang et al., 2018). Indeed, the PARP inhibitor olaparib has been shown to enhance the sensitivity of CC cells to CDDP (Prasad et al., 2017), substantiating the potential for miRNAs to provide new therapeutic targets.

#### Regulation of apoptosis

CDDP can promote cell death through both the external death receptor pathway or the internal mitochondrial pathway, providing a number of mechanisms by which interference with apoptosis might contribute to CDDP resistance. For example, miRNAs could upregulate pro-survival factors such as Bcl-2 and inhibitor of apoptosis protein, or inhibit the expression of tumor suppressor genes such as the caspases. Ectopic expression of miR-214 reduces the expression of the protective Bcl2-like 2 protein and increases the expression of the pro-apoptotic proteins Bax, caspase-9, caspase-8, and caspase-3 in CC cells, leading to enhanced apoptosis and CDDP sensitivity (Wang et al., 2013). Similarly, miRNA-218 can reduce the expression of survivin both in mRNA and protein level, a member of the inhibitor of apoptosis protein family, thereby increasing CDDP sensitivity in CC cells (Yu et al., 2019).

Alterations in signaling pathways that promote cell death can also lead to CDDP resistance, and blockade of these pathways with pharmacological inhibitors and/or RNA interference may have therapeutic effects. A number of miRNAs have been shown to contribute to CDDP resistance in CC cells through such mechanisms. Downregulation of the LDLR–PTEN pathway by miR-92b leads to activation of the AKT pathway in CC cells, which inhibits apoptosis (Sun et al., 2019). Overexpression of miR-218 inhibits proliferation and induces apoptosis in HeLa cells through inhibiting the AKT–mTOR signaling pathway (Li, Ping & Ning, 2012). PKC signaling is another key pathway for the regulation of chemosensitivity in cancer cells (Clark et al., 2003; Mohanty, Huang & Basu, 2005; Yang et al., 2016b). As noted earlier, miR-181a is a negative regulator of PRKCD and inhibits apoptosis in CC cells, thereby enhancing CDDP resistance (Chen et al., 2014). Taken together, these observations identify a large number of miRNAs and related signaling pathways that could serve as novel therapeutic targets to overcome CDDP resistance in CC.

#### Epithelial-mesenchymal transition

Epithelial-mesenchymal transition (EMT) is the process by which epithelial cells acquire a mesenchymal phenotype and is a critical element in the behavior of cancer cells, including chemotherapy resistance in CC (Goto et al., 2017; Li et al., 2018; Xiong et al., 2017). The crucial role played by miRNAs in regulating the EMT is considered to be another potential mechanism by which CC cells acquire CDDP resistance (Xu et al., 2017). CDDP-resistant CC cells display more EMT characteristics and increased migration and invasion compared with CDDP-sensitive cells (Song & Li, 2017). MiR-25-3p and miR-31-3p reverse the mesenchymal phenotype of CC cells by directly targeting semaphorin 4C, thus increasing CDDP sensitivity (Jing et al., 2019; Song & Li, 2017). MiR-20a mediates EMT that induced by protein phosphatase 1, regulatory subunit 13 like (PPP1R13L) and regulates CDDP resistance in HeLa cells (Xiong et al., 2017). In addition, CDDP sensitivity of CaSki cells is increased by treatment with epidermal growth factor, leading to upregulated miR-155 and reversal of the EMT (Lei et al., 2012). These results suggest that miRNAs regulating the EMT could be targets for reducing CDDP resistance in CC.

#### Targeting CSCs

As a rule, chemotherapy targets non-stem cell tumor cells, leaving an increased proportion of drug-resistant cancer stem cells (CSCs) in the tumor (Carnero et al., 2016; Leon et al., 2016; Nassar & Blanpain, 2016). Consequently, CSCs have become a potential target for therapeutic intervention for many cancers, including CC (Cao et al., 2017; Chhabra, 2015; Mendoza-Almanza et al., 2019). For example, miR-23b-mediated reduction of ALDH1A1 disturbs the homeostasis of cervical CSCs and promotes the CDDP sensitivity, overexpression of miR-23b re-sensitizes CC cells to CDDP treatment (Wang et al., 2017b).

#### Oxidative stress

The combination of CDDP and nucleophilic substances or thiol-containing proteins, such as glutathione and glutathione-S-transferase, can consume intracellular antioxidant reserves, thereby promoting oxidative stress, and excessive reactive oxygen species results in cell apoptosis or death (Lewis, Hayes & Wolf, 1988; Zhang et al., 2016). Thus, miR-497, which directly targets transketolase mRNA and promotes the generation of glutathione, reduces oxidant levels, and induces CDDP chemoresistance in CC cells (Yang et al., 2016a).

### The mechanisms by which lncRNAs affect CDDP resistance in CC

LncRNAs (>200 nucleotides) play important roles in gene transcription, protein translation, and chromatin remodeling (Kornienko et al., 2013). Not surprisingly, lncRNAs have been shown to be involved in many of the pathological behaviors of CC cells, including aberrant proliferation, migration, and invasion (Dong et al., 2017; Liang et al., 2019; Liu et al., 2018b; Zhu et al., 2018). Recent evidence has confirmed the roles of lncRNAs in CDDP resistance in CC (Iden et al., 2016; Wang et al., 2018).

LncRNAs can act as competitive endogenous RNAs (ceRNAs) by inhibiting the ability of miRNAs to interact with the same target mRNAs (Wilusz, Sunwoo & Spector, 2009; Yamamura et al., 2018; Yoon, Abdelmohsen & Gorospe, 2014). Alterations in critical interactions of the ceRNA regulatory network may thus affect CDDP resistance in CC. HOXD antisense growth-associated lncRNA (HAGLR) has been shown to act as a ceRNA for miR-130a-3p, thereby upregulating the expression of the miR-130a-3p target mRNA zinc finger E-box binding homeobox 1 (Chi et al., 2018). Similarly, crosstalk between the lncRNA NCK1-DT, miR-134-5p, and MutS protein homolog 2 has been shown to play a role in CDDP resistance (Zhang et al., 2019). The lncRNAs cancer susceptibility candidate 2 (CASC2) and growth arrest-specific 5 (GAS5) both act as ceRNAs for miR-21. CASC2 binding to miR-21 upregulates PTEN expression, reducing the phosphorylation of AKT (Feng et al., 2017); whereas GAS5 binding to miR-21 decreases CC cell apoptosis *via* STAT3 mRNA (Yao et al., 2019). LncRNA DANCR functions as a ceRNA for miR-665, which regulates ERK–SMAD signaling in CC cells (Cao et al., 2019).

LncRNAs also affect CDDP resistance by direct regulation of signaling pathway components. Knockdown of the lncRNA plasmacytoma variant translocation 1 (PVT1) in the human CC cell line SiHa upregulates the level of active caspase-3, which increases CDDP-induced apoptosis (Iden et al., 2016). Similarly, lncRNA taurine-upregulated gene 1 (TUG 1) promotes CDDP resistance in the disease progression of CC through activating the MAPK pathway (Wei et al., 2019). Besides, upregulation of lncRNA MALAT1 increases the expression of phosphorylated PI3K and AKT in HeLa and C-33A cells and promotes their CDDP resistance (Wang et al., 2018).

Many other ceRNA–miRNA–mRNA regulatory networks that contribute to CDDP resistance undoubtedly exist, and expanding our understanding of those mechanisms will provide more therapeutic opportunities for overcoming CDDP resistance.

### The mechanisms by which circRNAs affect CDDP resistance in CC

CircRNAs are endogenous ncRNAs which characterized by covalently closed loop without any 5′-3′ polarity or a polyadenylated tail (Chen & Yang, 2015). In recent years, increasing studies have found that circRNAs widespread expressed and played critical roles in biological processes such as tumor cell proliferation, apoptosis, invasion, and migration (Guo et al., 2014; Liu et al., 2018a; Memczak et al., 2013; Zhong et al., 2018). A study revealed 45 significantly highly expressed circRNAs in CC tissue through microarray analysis (Gao et al., 2017). Several studies have indicated that circRNAs were involved in CC development and progression by sponging miRNAs (Cai et al., 2019; Mao, Zhang & Li, 2019; Tang et al., 2019; Zhang et al., 2018b).

Since circRNAs can interact with miRNAs and modulate their expressions, they may also regulate the chemosensitivity of CDDP in CC. A study demonstrated that circMTO1 (mitochondrial translation optimization 1 homologue, ID: hsa_circ_0007874) could promote CC cell tumorigenesis and CDDP resistance through sponging miR-6893 (Chen et al., 2019). Another study identified hsa_circ_0023404 directly interacted with miR-5047 and inhibited autophagy-induced apoptosis to confer CDDP resistance of CC cells (Guo et al., 2019). CircRNAs play a significant role in chemoresistance of CC and may serve as future therapeutic biomarkers.

In addition to miRNAs, lncRNAs and circRNAs, piRNAs, as a new class of ncRNAs, have also shown a potential role in regulating CDDP resistance in CC. The upregulated expression of piR-651 was confirmed in HeLa cells, suggested that piRNAs may be involved in the development of CC (Cheng et al., 2011). Moreover, Hiwi, as a human homologue of the Piwi family and interacting with piRNAs, was found to be involved in the resistance of CC cells to CDDP (Liu et al., 2014). For most ncRNAs, our knowledge regarding their biological function is still limited, but their potential roles in pathogenesis and chemotherapy resistance of CC will be recognized in the future.

## Perspectives

CDDP effectively prolongs the survival of CC patients, but its efficacy and clinical application are limited by frequent emergence of drug resistance. As noted above, several ncRNAs associated with CDDP sensitivity/resistance of CC have been identified and are potential drug targets based on results of CC cell experiments (Cheng et al., 2018; Ping et al., 2018; Shih et al., 2019; Vella et al., 2015). Such ncRNAs may also be useful biomarkers to predict chemosensitivity and thus improve treatment regimens in this patient population.

The ncRNAs known to be differentially expressed in drug-resistant CC compared with normal tissues include miR-7-5p (Yang et al., 2018), miR-21( Feng et al., 2017), miR-130a (Feng et al., 2018), miR-181a (Chen et al., 2014), which are upregulated, and lncRNA CACS2, which is downregulated in CDDP-resistant CC (Feng et al., 2017). Analysis of the expression of ncRNAs in CC patients in combination with other molecular markers may predict the patient’s response and thus identify those who would most benefit from CDDP. The development of high-throughput technology makes the identification of molecular characteristics and genotypes more accurate and efficient, and will help to identify other differentially expressed ncRNAs with the potential to be predictive biomarkers.

The ncRNAs listed in Table 1 are associated with differential CDDP sensitivity, suggesting that ncRNA analogs or inhibitors may increase CDDP activity in humans, as has been demonstrated in cultured cells. Treatment of CDDP-resistant CC cells with a miR-25-3p mimic significantly sensitized them to growth inhibition by CDDP (Song & Li, 2017). Conversely, blockade of miR-7-5p with antisense oligonucleotide -miR-7-5p increased the apoptosis rate of CDDP-resistant CC cells (Yang et al., 2018). However, there have been no clinical trials or clinically relevant animal trials of these agents for CC to date. Adjuvant therapy with ncRNA-based agents may have advantages over other anti-cancer mechanisms, given that a single ncRNA can not only target multiple genes but also modulate resistance to multiple drugs. Nevertheless, there are also many challenges associated with the use of ncRNA-based agents as therapeutic drugs, including their stability in body fluids and tissues, drug delivery issues, and potential off-target effects that might result in an unfavorable safety profile.

**Table 1:** Recent advances of ncRNAs in cisplatin resistance for cervical cancer.

NcRNA	ID	Target	Effect on DDP sensitivity in CC	References
microRNA	miR-584	GLI1	increase	Wang, Feng & Zhang (2020)
microRNA	microRNA-708	Timeless	increase	Zou et al. (2020)
microRNA	microRNA-499a	SOX6	decrease	Chen et al. (2020)
microRNA	microRNA-138	H2AX	increase	Yuan et al. (2020)
microRNA	miR-21	SMAD7 unknown	decrease	Liu, Liu & Wang (2020) and Zhang et al. (2018a)
microRNA	miR-574-5p	QKI	increase	Tong et al. (2019)
microRNA	miR-218	Survivin AKT-mTOR signaling pathway	increase	Yu et al. (2019) and Li, Ping & Ning (2012)
microRNA	miR-217	KRAS	increase	Yin & Ren (2019)
microRNA	miR-31-3p	Sema4C	increase	Jing et al. (2019)
microRNA	miR-92b	LDLR, PTEN	decrease	Sun et al. (2019)
microRNA	miR-144	LHX2	increase	Shi et al. (2019)
microRNA	miR-1284	HMGB1	increase	Chen & Li (2018)
microRNA	miR-7-5p	PARP-1, BCL2	decrease	Yang et al. (2018)
microRNA	miR-130a	CTR1	decrease	Feng et al. (2018)
microRNA	miR-20a	iASPP	decrease	Xiong et al. (2017)
microRNA	miR-29b	STAT3 signaling pathway	increase	Li et al. (2017)
microRNA	miR-23b	ALDH1A1	increase	Wang et al. (2017b)
microRNA	miR-106a/b	SIRT1	decrease	Raji et al. (2017)
microRNA	miR-139-3p	Unknown	increase	Sannigrahi et al. (2017)
microRNA	miR-25-3p	Sema4C	increase	Song & Li (2017)
microRNA	miR-125a	STAT3	decrease	Fan et al. (2016)
microRNA	miR-497	TKT	decrease	Yang et al. (2016a)
microRNA	miR-182	PI3K/PTEN/AKT, PDCD4	decrease	Yang et al. (2016c)
microRNA	miR-183	KIAA1199, BAX	increase	Yang et al. (2016c)
microRNA	miR-30a	Beclin1	increase	Yang et al. (2016c)
microRNA	miR-664	E-Cadherin	increase	Yang et al. (2015b)
microRNA	miR-181a	PRKCD	decrease	Chen et al. (2014)
microRNA	miR-214	TFAM Bcl2l2	increase increase	Wen et al. (2014) and Wang et al. (2013)
microRNA	miR-155	TP53, SMAD2	increase	Lei et al. (2012)
microRNA	miR-199a	Unknown	decrease	Lee et al. (2008)
LncRNA	PCAT6	miR-543/ZEB1	decrease	Ma et al. (2020)
LncRNA	HNF1A-AS1	microRNA-34b/TUFT1	decrease	Luo et al. (2019)
LncRNA	DANCR	miR-665/TGFBR1, ERK/SMAD	decrease	Cao et al. (2019)
LncRNA	TUG1	RFX7, MAPK pathway	decrease	Wei et al. (2019)
LncRNA	ZFAS1	unknown	decrease	Feng et al. (2019)
LncRNA	NCK1-AS1	miR-134-5p/MSH2	decrease	Zhang et al. (2019)
LncRNA	GAS5	miR-21/STAT3	decrease	Yao et al. (2019) and Wen et al. (2017)
LncRNA	MALAT1	BRWD1, PI3K/AKT pathway	decrease	Wang et al. (2018)
LncRNA	HAGLR	miR-130a-3p, ZEB1	decrease	Chi et al. (2018)
LncRNA	UCA1	caspase-3, CKD2, surviving, p21	decrease	Wang et al. (2017a)
LncRNA	CASC2	miR-21, PTEN	increase	Feng et al. (2017)
LncRNA	PVT1	caspase-3	decrease	Iden et al. (2016)
CircRNA	circMTO1	miR-6893/S100A1	decrease	Chen et al. (2019)
CircRNA	circ_0023404	miR-5047/VEGFA and autophagy signaling pathway	decrease	Guo et al. (2019)

In order to solve the non-specific distributions of CDDP and the instability of miRNA in the systemic circulation, Wang and Liang used a liposome carrier to co-deliver two different therapeutics (miRNA-1284 and CDDP) into CC cells, and the carrier was specific towards its receptor overexpressed in CC cells, which resulted in enhanced accumulation of liposomes and increased chemosensitivity of CDDP (Wang & Liang, 2020). This study provides a new insight for ncRNA-based cancer therapy, and there are still many potential hurdles that need to be overcome before it can be tested clinically.

## Conclusions

In this review, we have summarized some of our current understanding of ncRNAs that affect CDDP sensitivity in CC. These studies have improved our understanding of the involvement of ncRNAs in drug resistance and provide a starting point for the development of agents to improve the efficacy of CDDP-based chemotherapy regimens and thus the quality of life and prognosis of CC patients.

## Additional Information and Declarations

### Data Availability

The following information was supplied regarding data availability: This is a literature review, and the research in this article did not generate any data or code.
